# Adolescent pregnancy and early gestation depressive symptoms in rural Bangladesh: Is there an association?

**DOI:** 10.1371/journal.pone.0317169

**Published:** 2026-03-12

**Authors:** Faysal Ahmed, Amena Al Nishan, Md. Alfazal Khan, Tahmeed Ahmed, S. M. Tafsir Hasan

**Affiliations:** 1 Nutrition Research Division, International Centre for Diarrhoeal Disease Research, Bangladesh (icddr, b), Dhaka, Bangladesh; 2 Health System and Population Studies Division, International Centre for Diarrhoeal Disease Research, Bangladesh (icddr, b), Dhaka, Bangladesh; 3 Office of Executive Director, International Centre for Diarrhoeal Disease Research, Bangladesh (icddr, b), Dhaka, Bangladesh; Mizan-Tepi University, ETHIOPIA

## Abstract

**Background/objective:**

Pregnancy during adolescence continues to pose a major public health concern in low-and middle-income countries, including Bangladesh. Suffering from depressive symptoms during pregnancy can lead to adverse maternal and fetal outcomes, and adolescent pregnancy may further complicate the risks. We aimed to estimate the prevalence of early-gestation depressive symptoms in pregnant adolescents in rural Bangladesh and investigate the potential association between the two.

**Methodology:**

Data for this study were extracted from the screening dataset of a community-based randomized controlled trial among pregnant women in Matlab, Bangladesh. Screening was conducted for all eligible expectant women in the study area after pregnancy was confirmed by ultrasound between 5–16 weeks of gestation. This study analyzed data from 651 newly pregnant adolescents and adult women screened between January 2020 and January 2021. Depressive symptoms were evaluated using the seven-item Depression subscale of the Depression, Anxiety, and Stress Scales-21 (DASS-21). Depressive symptom scores were calculated as the sum of the responses to the seven items, and a binary outcome, depressive symptoms, was defined as a depressive symptom score of 5 or more. Multivariable linear and log-binomial regression models were fitted to determine the independent association of depressive symptom score and depressive symptoms with adolescent pregnancy, considering adult pregnancy as the reference.

**Results:**

Of the pregnant women, 136 (20.9%) were adolescents (14–19 years), and 515 (79.1%) were adults (20–34 years). The prevalence of depressive symptoms in early gestation was found to be 11.0% among pregnant adolescent girls. In adjusted model, the mean depressive symptom score was higher among the adolescents compared to the adult pregnant women (β = 0.6, 95% CI: 0.1, 1.0; P = 0.010). In multivariable model, adolescents had higher prevalence ratio of depressive symptoms than their adult counterparts (PR = 2.0, 95% CI: 1.1, 3.6, P = 0.025).

**Conclusions:**

Our results suggest that adolescent pregnancy is associated with depressive symptoms in early gestation in rural Bangladesh. Furthermore, we found that more than one in ten pregnant adolescents experiences symptoms of depression in the early stages of pregnancy, underscoring a serious public health issue. Public health policies should aim at preventing adolescent marriage and pregnancy in the first place, and programs should be launched to mitigate depressive symptoms among pregnant adolescents.

## Introduction

Adolescent pregnancy remains a major public health concern globally, with deleterious consequences for both maternal and fetal outcomes. Although the global adolescent birth rate has slightly decreased, regional disparities persist, with higher burdens in low- and middle-income countries (LMICs) [1]. Approximately 21 million teenage girls from LMICs become pregnant every year [1]. Bangladesh has the highest rate in Asia, with 74 births per 1,000 girls aged 15–19 years [2].

Adolescence is a critical life transition phase characterized by significant biological, psychological, and social changes with lifelong impacts [3]. Pregnancy, another life-altering phase, introduces dramatic changes to the body’s complex physiological, hormonal, and psychological balance [4,5]. When pregnancy occurs during adolescence, it presents unique challenges and risks that may adversely affect the individual’s overall health and well-being. The consequences of adolescent pregnancy are severe, ranging from increased risk of abortion, stillbirth, preterm birth, low birth weight, malnutrition, and infections to maternal and neonatal death [6]. Additionally, adolescent pregnancy adversely affects individuals financially, socially, mentally, and emotionally [7,8]. Moreover, antenatal depression poses risks of adverse maternal and fetal outcomes [9–11], with first-trimester depression in pregnancies often being more severe [12]. The already complex and high-risk nature of adolescent pregnancy becomes even more challenging if it is again layered with depression. The existing burden of poverty, poor living conditions, inadequate education, chronic infections, and malnutrition may further complicate the overall health and mental state of pregnant teenagers in LMICs [13–15].

Several studies have reported a high prevalence of antenatal depressive symptoms among pregnant adolescents [16,17]. In a study conducted in Nairobi, Kenya, 43.1% of 153 pregnant adolescents reported suffering from depressive symptoms, of whom 16.3% were in their first trimester [17]. Another study conducted in Thailand reported 46% prevalence of depression among pregnant teenagers measured by the Edinburgh Postnatal Depression Scale (EPDS) questionnaire for depression screening [16]. In a recent literature review, Lesinskienė et al. also emphasized the increased incidence of depressive symptoms among adolescent pregnant mothers [18].

The combined and consequential risks of pregnancy during adolescence can be particularly severe when accompanied by depressive symptoms. Undiagnosed and untreated prenatal depressive symptoms can lead to reluctance to seek proper antenatal care, postpartum depression, and other adverse consequences for maternal and fetal health [19,20]. Early detection of antenatal depression facilitates timely intervention, ensures appropriate management, and helps mitigate its adverse effects [20,21]. Although data exist on antenatal depressive symptoms in adult women [22] and on postpartum depression [23] in Bangladesh, information on prenatal depressive symptoms, especially during early gestation in pregnant adolescents in Bangladesh, remains limited. Furthermore, it is also important to examine whether there is a positive association between adolescent pregnancy and depressive symptoms in early gestation. This study aimed to estimate the prevalence of early-gestation (5–16 weeks) depressive symptoms in pregnant adolescents in rural Bangladesh and investigate the potential association between the two.

## Methods

### Study setting, population, and data source

This analysis used data from the screening dataset of a community-based randomized controlled trial entitled ‘Improving Maternal Nutrition in Matlab (IMNiM)’ (NCT04868669) conducted in Matlab, a South-Eastern rural subdistrict of Bangladesh. Details of the screening activity and the study setting have been described elsewhere [24,25]. In brief, this trial screened 683 successively reported and ultrasound-confirmed newly pregnant women in their early gestation (5–16 weeks) from 14 January 2020 to 31 January 2021 amid the COVID-19 pandemic. The data were accessed for this particular analysis on 8 January 2024.

The study area is part of the Matlab Health and Demographic Surveillance System (HDSS) maintained by the International Centre for Diarrhoeal Disease Research, Bangladesh (icddr,b), which enables systematic identification of pregnancies and facilitates early access to antenatal care (ANC) [26]. Community Health Research Workers (CHRWs), appointed by icddr,b, identify potential new pregnancies based on the history of amenorrhea, followed by urine strip tests in some cases. Women residing in the icddr,b service area of HDSS participated in the screening activity of the IMNiM trial. The screening took place at Matlab Hospital, where two-thirds of pregnant women from this rural area seek ANC during early gestation at Matlab Hospital [26]. Women were approached for screening if they were found pregnant on ultrasound examination.

The screening process included anthropometric measurements, assessment of depressive symptoms (as depressive symptoms were part of the exclusion criteria for the main trial), household food insecurity, and data collection on chronic diseases, pregnancy complications, obstetric history, and sociodemographic variables through a pretested semi-structured questionnaire. Obstetric and clinical data were documented following consultations with on-duty physicians at Matlab Hospital. The accurate ages of the women were retrieved from the HDSS database.

While the main study is a prospective, two-arm, parallel group, cluster-randomized controlled trial, this analysis is based solely on the screening data collected prior to randomization and intervention allocation. Therefore, this study followed a cross-sectional design.

Advanced-age pregnancies (women older than 35 years) were excluded from the screening dataset, and this analysis was limited to 651 pregnant adolescent girls and adult pregnant women. We categorized participants into adolescents (<20 years, n = 136) and adults (20–35 years, n = 515) based on age and compared these two groups to explore differences in early gestation depressive symptoms. Although the number of adolescents was relatively smaller, it reflected the actual age distribution of pregnancies in the screening dataset and the catchment community. As we approached all pregnant women in the study area for screening and the refusal for screening was very low (about 1.8%), the screening dataset offers representative insights into this rural context.

### Adolescent pregnancy and comparator

In our study, pregnant adolescent girls were defined as those who became pregnant between the ages of 14 and 19 years [27]. Comparators were adult pregnant women aged 20–35 years. Women older than 35 years were excluded because advanced-age pregnancies are associated with adverse perinatal and mental health outcomes [28–30].

### Outcome

The outcome of interest was depressive symptoms in early gestation. Depressive symptoms in pregnant adolescent girls and adult pregnant women over the past week were assessed using the depression subscale of the Depression, Anxiety, and Stress Scales-21 (DASS-21) [31,32], which has been validated for use in both clinical and non-clinical populations [33–37].

The DASS-21 depression subscale consists of 7 items that measure depressive symptoms. Responses are scored on a 4-point Likert scale ranging from 0 (did not apply to me at all) to 3 (applied to me very much/most of the time), with higher scores indicating higher levels of depressive symptoms. The total score for the depression subscale ranges from 0 to 21, with a score of 5 or above suggesting the presence of depressive symptoms [31]. The DASS-21 depression subscale has demonstrated good convergent validity with the Beck Depression Inventory (r = 0.74) [32,38,39] and excellent internal consistency (Cronbach’s alpha = 0.81) [32,39–41].

In this study, the validated Bengali version of the DASS-21 [42–44] was used. The tool was contextualized to ensure that all statements were culturally appropriate and easily understood by pregnant adolescent girls and adult women after being piloted with 22 adolescent girls and adult women [24]. The DASS-21 is a self-report instrument that does not require any special skills or training to administer. Pregnant adolescent girls and adult pregnant women read and recorded their responses themselves; however, for a few individuals who could not read Bengali, a trained interviewer read the statements aloud and recorded their responses.

This study examined the presence of depressive symptoms both as a continuous measure (depressive symptom score) and as a binary variable (depressive symptoms defined as a score of 5 or more in the DASS-21 depression subscale).

### Covariates

Guided by previous literature [13,24,45–47] and the availability of data, we considered nutritional status, gestational age, nulliparity, pregnancy complications, chronic diseases, religion, education, and household food insecurity as potential covariates. Nutritional status was assessed using early gestation BMI (<18.5 kg/m² underweight, 18.5– < 25 normal, ≥ 25 overweight/obese) [48], and further dichotomized as normal versus abnormal, with the abnormal category comprising underweight and overweight/obese participants. Weight and height were measured with calibrated equipment (Tanita HD‑661 scale; Seca 213 stadiometer). Gestational age was calculated from the last menstrual period and confirmed by ultrasound [49]; nulliparity was defined as no previous live birth [50]. Pregnancy complications and chronic diseases encompass common obstetric and medical conditions, respectively. Education was categorized as primary (≤5 years), some secondary (6–9 years), and secondary completed (≥10 years). Household food insecurity was measured using the Household Food Insecurity Access Scale (HFIAS) [51], translated and piloted locally [24]; due to small subgroup sizes, mild, moderate, and severe categories were combined into a single ‘food insecure’ group.

### Statistical analysis

We summarized the sociodemographic, anthropometric, clinical, and pregnancy-related characteristics of pregnant adolescent girls and adult pregnant women in the sample as percentages for categorical variables and mean (standard deviation) for continuous variables. We compared the characteristics between the two groups using the chi-square test for categorical variables and the t-test for continuous variables.

In our analysis, we used the seven items comprising the depression subscale of the Depression Anxiety Stress Scales (DASS-21). Following the official DASS-21 manual [52–55], we summed the seven item scores to generate the total depression score. Although individual Likert-type items like the DASS-21 are ordinal in nature, it is standard and widely accepted practice to treat the summed DASS-21 subscale scores as a continuous measure of the underlying construct. This approach has been extensively applied and validated in prior research [53–56]. While statistical analyses that treat single ordinal items as continuous may be inappropriate, the aggregation of multiple ordinal items into a composite score representing a latent construct (such as depressive symptoms) is commonly accepted as a reasonable approximation of a continuous variable [57–59]. We visualized the bivariate relationship between the depressive symptom score and the age of the participants through a scatterplot with superimposed regression lines, separately fitted for pregnant adolescent girls and adult pregnant women.

We examined the association of depressive symptom score with adolescent pregnancy through simple and multiple linear regression, using adult pregnant women as the reference group. We expressed the strength of association as mean difference (β) with a 95% confidence interval (95% CI). We also used Rasch-modeled scores [60] for sensitivity analyses, with findings presented in the supplementary materials. The Rasch model transforms ordinal responses into interval-level estimates of latent traits (logit scores) that account for item difficulty and response thresholds [60]. To assess the association of adolescent pregnancy with depressive symptoms (binary variable), simple and multivariable log-binomial regression models were fitted. We expressed the strength of association as prevalence ratio (PR) with 95% CI, considering adult pregnant women as the reference group. For sensitivity analysis, we fitted Poisson regression models with robust variance as an alternative to log-binomial regression, which is recommended for handling convergence issues and improving the stability of estimates in situations with outcome imbalance or unequal group sizes [61,62].

All the covariates of a priori interest such as nutritional status, gestational age, nulliparity, pregnancy complications, chronic diseases, religion, education, and household food insecurity were initially considered for inclusion in the multivariable regression models; however, nulliparity was removed from the final models due to multicollinearity with the primary explanatory variable (pregnant adolescent girls or adult pregnant women). All statistical tests were two-sided, and statistical significance was evaluated at p < 0.05. Statistical analyses were performed with Stata/PC (StataCorp, College Station, Texas 77845 USA, version 17.0).

### Ethical consideration

The study was conducted in accordance with the guidelines of the Declaration of Helsinki. The study (Protocol ID: PR-19109) was approved by the Research Review Committee and the Ethical Review Committee (Institutional Review Board) of icddr,b. Verbal informed consent was obtained from all women undergoing screening in the presence of a witness. For pregnant adolescent girls under 18 years, consent was obtained from their legal guardians. Consenting individuals were informed that identity and personal health information would remain confidential, but the data might be analyzed and published anonymously. They were assured that participation was voluntary and that refusal to participate in screening would not affect the care provided by icddr,b.

The consent-taking procedure was approved by the Institutional Review Board of icddr,b as part of the protocol, ensuring that no identity-related or personal health information would be disclosed and that only aggregated data would be reported for scientific purposes. The Ethical Review Committee does not require separate approval or documentation of verbal consent for the use of anonymized screening data for research purposes.

## Results

Of the final sample, 136 (20.9%) were pregnant adolescent girls, and 515 (79.1%) were adult pregnant women. The sample characteristics are provided in Table 1.

**Table 1:** Sample characteristics by age group.

	Overall n = 651	Pregnant adolescents n = 136	Pregnant women n = 515	p-value
Age (years), Mean (SD)	24.1 (5.1)	17.8 (1.1)	25.8 (4.4)	<0.001
Nutritional status				<0.001
Underweight	95 (14.6)	34 (25.0)	61 (11.8)	
Normal	398 (61.1)	90 (66.2)	308 (59.8)	
Overweight/obese	158 (24.3)	12 (8.8)	146 (28.4)	
Gestational age (weeks), Mean (SD)	9.8 (1.8)	9.9 (2.0)	9.8 (1.7)	0.719
Nulliparity	237 (36.4)	116 (85.3)	121 (23.5)	<0.001
Pregnancy complications	47 (7.2)	5 (3.7)	42 (8.2)	0.073
Chronic diseases	34 (5.2)	6 (4.4)	28 (5.4)	0.633
Religion				0.695
Muslim	548 (84.2)	113 (83.1)	435 (84.5)	
Hindu	103 (15.8)	23 (16.9)	80 (15.5)	
Education (years)				<0.001
Primary	82 (12.6)	2 (1.5)	80 (15.5)	
Some secondary	324 (49.8)	85 (62.5)	239 (46.4)	
Secondary completed	245 (37.6)	49 (36.0)	196 (38.1)	
Household food insecurity	66 (10.1)	12 (8.8)	54 (10.5)	0.568

All values are n (%) unless otherwise indicated.

The mean (SD) age of the pregnant adolescents was 17.8 (1.1) years and that of the adult women was 25.8 (4.4) years. Nutritional status was significantly different between the two groups (p < 0.001): 25.0% of the adolescents were underweight, while 11.8% of the adult women were underweight. Of the adolescents, 85.3% were nulliparous, while nulliparity was found among 23.5% of the adult women. The overall mean (SD) gestational age was 9.8 (1.8) weeks.

In the study, the overall mean (SD) depressive symptom score was 1.2 (2.2). The prevalence of depressive symptoms in early gestation was 11.0% and 6.6% among pregnant adolescent girls and adult pregnant women, respectively (Table 2).

**Table 2:** Depressive symptoms among pregnant adolescent girls and adult pregnant women in early gestation (n = 651).

	Overall n = 651	Pregnant adolescents n = 136	Pregnant women n = 515
Depressive symptom score, Mean (SD)	1.2 (2.2)	1.6 (2.8)	1.1 (2.0)
Depressive symptoms, % (95% CI)	7.5 (5.7, 9.5)	11.0 (6.6, 17.1)	6.6 (4.7, 9.0)

**Fig 1:**
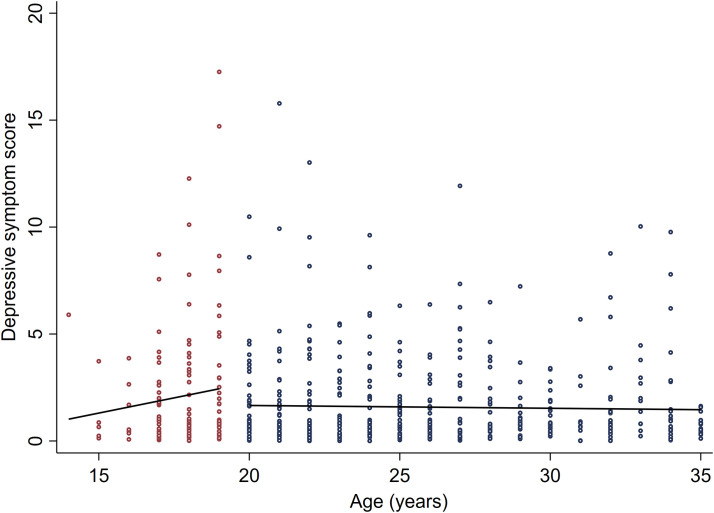
Association between depressive symptom score and age among pregnant adolescent girls and adult pregnant women. Circles indicate depressive symptom scores (with a jitter effect to prevent overlapping) and straight lines indicate fitted lines through linear regression between age and depressive symptom scores separately for adolescents and adult women.

Fig 1 visualizes the bivariate relationship between depressive symptom score and age among pregnant adolescent girls and adult pregnant women. There appears to be an increasing trend in depressive symptom scores with age among adolescents, while the trend is flat to minimally decreasing among adult women.

Table 3 shows the association of adolescent pregnancy with depressive symptom score in early gestation. In the adjusted model, pregnant adolescent girls had higher mean depressive symptom score compared to their adult counterparts (β = 0.6, 95% CI: 0.1, 1.0; p = 010).

**Table 3:** Association between adolescent pregnancy and depressive symptom scores in early gestation using linear regression (n = 651).

Depressive symptom score	Unadjusted		Adjusted*	
**ß (95% CI)**	**p-value**	**ß (95% CI)**	**p-value**
0.5 (0.1, 0.9)	0.025	0.6 (0.1, 1.0)	0.010

*Adjusted for nutritional status, gestational age, pregnancy complications, chronic diseases, education, religion, and household food insecurity. (R² = 0.0514 for adjusted model)

A similar association between adolescent pregnancy and depressive symptom score was also found in the adjusted Rasch-estimated linear regression model. There was no discernible change in effect direction or statistical significance (S1 Table).

Table 4 shows the prevalence ratio of depressive symptoms in early gestation among pregnant adolescent girls compared to their adult counterparts. In the adjusted model, the prevalence ratio of depressive symptoms was higher in pregnant adolescents (PR = 2.0, 95% CI: 1.1, 3.6; p = 0.025).

**Table 4:** Prevalence ratio of depressive symptoms in early gestation among pregnant adolescent girls compared to adult pregnant women using log-binomial regression (n = 651).

Depressive symptoms	Unadjusted		Adjusted*	
	PR (95% CI)	p-value	PR (95% CI)	p-value
1.7 (0.9, 3.0)	0.081	2.0 (1.1, 3.6)	0.025

*Adjusted for nutritional status, gestational age, pregnancy complications, chronic diseases, education, religion, and household food insecurity.

The multivariable Poisson regression with robust variance also suggests a similar association between adolescent pregnancy and depressive symptoms. We found no discernible change in effect direction or statistical significance (S2 Table).

## Discussion

Our study reveals that 11% of pregnant adolescent girls in rural Matlab experience depressive symptoms during the first trimester of pregnancy. Although no prior studies in Bangladesh have reported depressive symptom prevalence among pregnant adolescents during the first trimester, this rate is somewhat comparable to findings from a study in two rural subdistricts of Mymensingh, where 16.7% of pregnant women under 20 reported antepartum depressive symptoms during the third trimester [22].

Our findings demonstrate that adolescent pregnancies are significantly associated with a higher prevalence of depressive symptoms during the first trimester, with adolescents being twice as likely to experience depressive symptoms compared to adult pregnant women. A recent study conducted in Bangladesh, covering both urban and rural areas, reported an elevated rate of suicide attempts during the postpartum period among adolescent mothers [63]. This finding somewhat aligns with our findings, as suicidal tendencies reflect deteriorated mental health and depressive symptoms [64]. Similar results from other LMICs further emphasize the burden of depressive symptoms linked to adolescent pregnancies. For example, studies in Kenya [17] and Ethiopia [65] reported a 43.1% and 37.4% prevalence of depressive symptoms among adolescent mothers during the antepartum and postpartum periods, respectively. While these studies did not assess depressive symptoms during early pregnancy, they underscore the pervasive mental health challenges faced by adolescent mothers in LMICs.

Interestingly, we observed a rising trend in depressive symptoms scores with age among pregnant adolescents, in contrast to a flat-to-declining trend observed among adult women. This highlights the heightened vulnerability of late teenage mothers to depressive symptoms. One possible explanation could be that individuals undergo emotional and cognitive shifts during adolescence [66], with late teens potentially being more aware of their surroundings and better able to articulate their emotions compared to early teens [67]. However, this interpretation contrasts with findings from Pereira et al., who reported more intense depression among younger adolescents in Brazil [68]. Further research is necessary to clarify this complex relationship.

Antenatal depressive symptoms during pregnancy have serious consequences for both the mother and the offspring, both during and after the pregnancy period. Moreover, depressive symptoms in the first trimester of adolescent pregnancy tend to be more severe compared to other trimesters [69]. Untreated first-trimester depressive symptoms may progress to postpartum depressive symptoms, potentially persisting lifelong [12]. Prenatal depressive symptoms can lead to unwillingness to seek proper antenatal care, poor gestational weight gain, malnutrition, preeclampsia, low birth weight, preterm birth, increased need for surgical interventions, poor neonatal outcomes, and higher rates of admission to neonatal intensive care units [9,10]. The high prevalence of antenatal depressive symptoms among adolescents underscores the double burden of adverse maternal and child health outcomes, emphasizing the need for public health programs tailored to teenagers and teenage mothers.

Although teenage pregnancy is a global problem, the prevalence of adolescent motherhood is significantly higher in resource-poor LMICs compared to high-income countries [70,71]. In developed regions, adolescent pregnancy is often viewed as a social issue, with most teenage mothers being unmarried [72]. Evidence from developed countries indicates a link between adolescent substance use and sexual risk behaviors, suggesting that teenage pregnancy is interconnected with illicit drug abuse and lack of safe contraception [73,74]. In contrast, in LMICs, teen marriage is generally culturally accepted in low-income communities, and teenage pregnancies usually occur within marital contexts [75]. Approximately 90% of adolescent motherhood in LMICs occurs among married teenage girls [76]. Bangladesh is particularly burdened by one of the highest prevalence of child marriage globally [77] and also has one of the highest adolescent fertility rates [2,78]. Factors such as low socioeconomic status, limited education, social norms and rituals, and religious beliefs contribute to child marriages and, consequently, teenage pregnancies [79–81]. Interventions targeting these factors could help reduce adolescent pregnancies, which, in turn, would minimize rates of early-gestation depressive symptoms among teenage mothers. Strategic policies should be implemented in rural settings to reduce girl-child marriage and subsequent adolescent pregnancies in Bangladesh, with special attention to cultural and social norms as well as entrenched religious superstitions. Additionally, early detection of depressive symptoms and timely intervention can help prevent further progression and adverse outcomes. In a country like Bangladesh, where mental health issues are often neglected, our study highlights the necessity of well-planned and tailored programs to address mental health, specifically among pregnant teenagers in early gestation.

To the best of our knowledge, this study is the first to investigate the association between adolescent pregnancy and early-gestation depressive symptoms in a rural setting in Bangladesh. A key strength of our study is the robust multivariate analysis to assess this association, adjusting for several high-priority confounders. However, the cross-sectional design limits causal inference, underscoring the need for longitudinal research to explore the trajectory of depressive symptoms from early pregnancy to the postpartum period. The unequal distribution of adolescent and adult pregnant women in this study may have influenced the precision of the estimated associations. To address this, we employed modified Poisson regression models as sensitivity analyses; however, the imbalance should be considered when interpreting the results. Moreover, the overall prevalence of antenatal depressive symptoms observed in our study was 7.5%, which is lower than the rates reported in other Bangladeshi studies, ranging from 18.3% to 56.6% [22,82,83]. Unlike these studies, which were conducted either during the third trimester [22,82] or across all three trimesters [83], our study focused exclusively on the first trimester depressive symptoms, which potentially explain the lower prevalence. Additionally, variability in findings may arise from differences in the screening tools employed, as the accuracy of self-reported depression screening tools can produce results sensitive to individual-specific factors [84]. Other factors contributing to the variations may include differences in study settings, sociodemographic characteristics, methodological approaches, and sample sizes. It is important to acknowledge that our study may have underestimated the prevalence of depressive symptoms. This underestimation could stem from the fact that we examined only one rural area and included households that reported pregnancies. Additionally, the data collection occurred during the COVID-19 pandemic, when limited access to the internet and mobile phones in resource-poor rural settings may have systematically excluded pregnancies from poorer households.

## Conclusions

Our findings indicate that adolescent pregnancy is correlated with depressive symptoms during early gestation in rural Bangladesh. Additionally, we found that more than one in ten pregnant adolescents suffers from depressive symptoms in early gestation. Public health policies should prioritize preventing adolescent marriage and pregnancy, while programs are needed to address depressive symptoms among pregnant adolescents. These programs should address cultural norms, offer mental health support, and promote education. In a context where mental health is often overlooked, this study underscores the urgent need for tailored mental health care for adolescent mothers during early pregnancy.

## Supporting information

S1 Table Association between adolescent pregnancy and Rasch-estimated latent depressive symptom scores in early gestation using linear regression (n = 651). (DOCX)

S2 Table Prevalence ratio of depressive symptoms (binary) in early gestation among pregnant adolescent girls compared to adult pregnant women using Poisson regression with robust variance (n = 651). (DOCX)
