# Supplementary material for: Adolescent pregnancy and early gestation depressive symptoms in rural Bangladesh: Is there an association?
Source: PLoS One. 2026 Mar 12;21(3):e0317169. doi: 10.1371/journal.pone.0317169 (PMC12981501; doi:10.1371/journal.pone.0317169)
Supplement: S1 Table — (DOCX) [file pone.0317169.s001.docx]

**S1 Table. Association between adolescent pregnancy and Rasch-estimated latent depressive symptom scores in early gestation using linear regression (n = 651)**

|  | **Unadjusted** | | **Adjusted*** | |
| --- | --- | --- | --- | --- |
| **Factors** | **ß (95% CI)** | **p-value** | **ß (95% CI)** | **p-value** |
| **Participant’s age** |  | |  |  |
| Adolescent (14-19 Years) | 0.12 (-0.01, 2.6) | 0.073 | 0.2 (0.02, 0.3) | 0.022 |
| Adult (20-35Years) | ref |  | ref |  |
| **Chronic diseases** |  | |  |  |
| Yes | 0.4 (0.1, 0.6) | 0.002 | 0.4 (0.2, 0.7) | <0.001 |
| No | ref |  | ref |  |
| **Pregnancy complications** |  | |  |  |
| Yes | 0.1 (-0.07, 0.4) | 0.194 | 0.1 (-0.1, 0.4) | 0.191 |
| No | ref |  | ref |  |
| **Nutritional status** |  | |  |  |
| Not normal  (Underweight/Overweight/Obese) | 0.1 (-0.05, 0.2) | 0.249 | 0.1 (-0.04, 0.2) | 0.206 |
| Normal | ref |  | ref |  |
| **Religion** |  | |  |  |
| Hindu | -0.07 (-0.2, 0.1) | 0.316 | -0.1 (-0.2, 0.1) | 0.449 |
| Muslim | ref |  | ref |  |
| **Education (completed years)** |  | |  |  |
| Secondary completed  or more (10 or more) | 0.03 (-0.2, 0.2) | 0.783 | 0.1 (-0.1, 0.3) | 0.397 |
| Secondary (6-9 years) | -0.1 (-0.3, 0.1) | 0.374 | -0.1 (-0.2, 0.1) | 0.511 |
| Primary or less (0-5 years) | ref |  | ref |  |
| **Gestational age (weeks)** | -0.03 (-0.1, 0.0004) | 0.174 | -0.02 (-0.1, 0.01) | 0.174 |
| **Household food insecurity** |  | |  |  |
| Food insecure | 0.4 (0.2, 0.6) | <0.001 | 0.4 (0.2, 0.6) | <0.001 |
| Food secure | ref |  | ref |  |

*Adjusted for nutritional status, gestational age, pregnancy complications, chronic diseases, education, religion, and household food insecurity.
