# Supplementary material for: Adolescent pregnancy and early gestation depressive symptoms in rural Bangladesh: Is there an association?
Source: PLoS One. 2026 Mar 12;21(3):e0317169. doi: 10.1371/journal.pone.0317169 (PMC12981501; doi:10.1371/journal.pone.0317169)
Supplement: S2 Table — (DOCX) [file pone.0317169.s002.docx]

**S2 Table. Prevalence ratio of depressive symptoms (binary) in early gestation among pregnant adolescent girls compared to adult pregnant women using Poisson regression with robust variance (n = 651)**

|  | **Unadjusted** | | **Adjusted*** | |
| --- | --- | --- | --- | --- |
| **Factors** | **PR (95% CI)** | **p-value** | **PR (95% CI)** | **p-value** |
| **Participant’s age** |  | |  |  |
| Adolescent (14-19 Years) | 1.7 (0.9, 3.0) | 0.082 | 1.8 (1.004, 3.3) | 0.048 |
| Adult (20-35Years) | ref |  | ref |  |
| **Chronic diseases** |  | |  |  |
| Yes | 3.0 (1.5, 6.2) | 0.003 | 3.5 (1.7, 7.2) | <0.001 |
| No | ref |  | ref |  |
| **Pregnancy complications** |  | |  |  |
| Yes | 1.5 (0.6, 3.5) | 0.397 | 1.4 (0.6, 3.3) | 0.480 |
| No | ref |  | ref |  |
| **Nutritional status** |  | |  |  |
| Not normal  (Underweight/Overweight/Obese) | 1.5 (0.9, 2.6) | 0.133 | 1.7 (0.9, 2.9) | 0.074 |
| Normal | ref |  | ref |  |
| **Religion** |  | |  |  |
| Hindu | 0.9 (0.4, 1.9) | 0.760 | 0.9 (0.4, 1.9) | 0.769 |
| Muslim | ref |  | ref |  |
| **Education (completed years)** |  | |  |  |
| Secondary completed  or more (10 or more) | 1.9 (0.7, 5.4) | 0.214 | 2.3 (0.8, 6.1) | 0.107 |
| Secondary (6-9 years) | 1.4 (0.5, 3.9) | 0.532 | 1.4 (0.5, 3.9) | 0.555 |
| Primary or less (0-5 years) | ref |  | ref |  |
| **Gestational age (weeks)** | 1.01 (0.9, 1.2) | 0.945 | 1.04 (0.9, 1.2) | 0.638 |
| **Household food insecurity** |  | |  |  |
| Food insecure | 2.0 (1.01, 3.9) | 0.046 | 2.3 (1.2, 4.4) | 0.015 |
| Food secure | ref |  | ref |  |

*Adjusted for nutritional status, gestational age, pregnancy complications, chronic diseases, education, religion, and household food insecurity.
